# Two Cases of Inguinal Hernia after Kidney Transplantation Treated with Transabdominal Preperitoneal

**DOI:** 10.70352/scrj.cr.25-0597

**Published:** 2026-01-16

**Authors:** Hitomi Zotani, Tetsu Yamamoto, Ryoji Hyakudomi, Kiyoe Takai, Takahito Taniura, Kazunari Ishitobi, Keisuke Inoue, Shunsuke Kaji, Takeshi Matsubara, Masaaki Hidaka

**Affiliations:** 1 Department of Digestive and General Surgery, Shimane University Faculty of Medicine, Izumo, Shimane, Japan; 2 Department of Surgery, Oda Municipal Hospital, Oda, Shimane, Japan; 3 Department of Surgery, Unnan City Hospital, Unnan, Shimane, Japan; 4 Department of Surgery, Matsue Red Cross Hospital, Matsue, Shimane, Japan

**Keywords:** kidney transplantation, inguinal hernia, TAPP, laparoscopic surgery

## Abstract

**INTRODUCTION:**

The Lichtenstein procedure is often selected for kidney transplantation (KT) recipients due to concerns about the risk of injury to the transplanted organ. However, there have been reports of complications such as ureteral obstruction in cases where the Lichtenstein procedure was performed; therefore, safer techniques and approaches must be selected. We herein report 2 cases of inguinal hernia on the transplant side after KT that were treated with laparoscopic hernia repair (transabdominal preperitoneal [TAPP]) following a careful preoperative assessment.

**CASE PRESENTATION:**

The first case was a 55-year-old man who had undergone KT 9 months earlier and developed a bulge in the right inguinal region 3 months prior to presentation. Contrast-enhanced CT was used to assess the ureteral course, and the extent of TAPP dissection was determined. Intraoperative findings revealed a direct hernia. Dissection was performed while confirming the ureteral course, and TAPP repair was completed. Postoperatively, the patient’s condition progressed uneventfully without recurrence at 21 months. The second case was a 58-year-old woman who had undergone KT 1 year earlier and subsequently developed swelling in the right inguinal region. Due to deterioration of the kidney function, enhanced CT was to be avoided. Plain CT was thus performed, but the evaluation of the transplanted ureter course was difficult. Laparoscopic observation was performed to evaluate the location of the transplanted ureter and kidney. A direct hernia was recognized. After determining the dissection of exposure of the myopectineal orifice and mesh deployment, TAPP repair was performed completely. Postoperatively, the patient’s condition progressed uneventfully without recurrence at 14 months.

**CONCLUSIONS:**

A preoperative evaluation of the transplanted kidney and ureter using CT is an important surgical strategy for KT patients with groin hernias. The TAPP procedure is appropriate because it allows safe visualization of the hernia type and the transplanted ureter from the abdominal cavity and enables dissection. TAPP was considered the most appropriate surgical procedure when an adequate distance between the hernial orifice and transplanted ureter could be confirmed.

## Abbreviations

BUN
: blood urea nitrogen
CE-CT
: contrast-enhanced CT
eGFR
: estimated glomerular filtration rate
HIPA
: high-incision peritoneal approach
KT
: kidney transplantation
MPO
: myopectineal orifice
TAPP
: transabdominal preperitoneal

## INTRODUCTION

The frequency of kidney transplantation (KT) is increasing in Japan, with 2001 cases per year in 2023.^1)^ Accordingly, the number of surgeries for KT recipients is increasing gradually, and inguinal hernia is a reported complication after KT.^1)^ Although surgery is the first choice of treatment for inguinal hernias, laparoscopic surgery tends to be avoided in KT recipients. The reasons for this are concerns regarding ureteral compression and blood flow suppression due to pneumoperitoneum. There is a risk of injury to the transplanted ureter because it runs around the hernial orifice. Thus, the Lichtenstein procedure, which does not require preperitoneal dissection, tends to be preferred.^2,3)^ However, some studies have reported complications after herniorrhaphy with the Lichtenstein procedure in KT recipients. Hence, safer techniques and approaches need to be developed and selected.^4–6)^

We herein report 2 cases of ipsilateral inguinal hernia after KT that were treated safely using the transabdominal preperitoneal (TAPP) procedure with a careful preoperative examination.

## CASE PRESENTATION

### Case 1

#### Chief complaints

A 55-year-old man with a right inguinal bulge was referred to our department.

#### Details of the present illness

The patient’s symptoms started 3 months before the consultation, and gradually exacerbated to the point of pain.

#### The history of past illnesses

He had been introduced to dialysis at 21 years old due to kidney failure caused by acquired cystic disease of the kidney. He underwent deceased-donor KT at 54 years old (9 months before his first visit).

#### Physical examination findings

His height was 178.8 cm, and his body weight was 61.85 kg. On physical examination, the abdomen was flat and soft, and a surgical scar (approximately 15 cm) from the Gibson incision for KT was observed in the right lower quadrant. A bulge the size of a tennis ball was noted in the right inguinal area while standing, which spontaneously disappeared on lying down.

#### Laboratory examinations

A blood analysis showed blood urea nitrogen (BUN), creatinine, and estimated glomerular filtration rate (eGFR) values of 19.6 mg/dL, 1.23 mg/dL, and 49.0 mL/min/1.73 m^2^, respectively, suggesting mild kidney impairment. However, no inflammation or other abnormalities were observed.

#### Imaging examinations

**Fig. 1:**
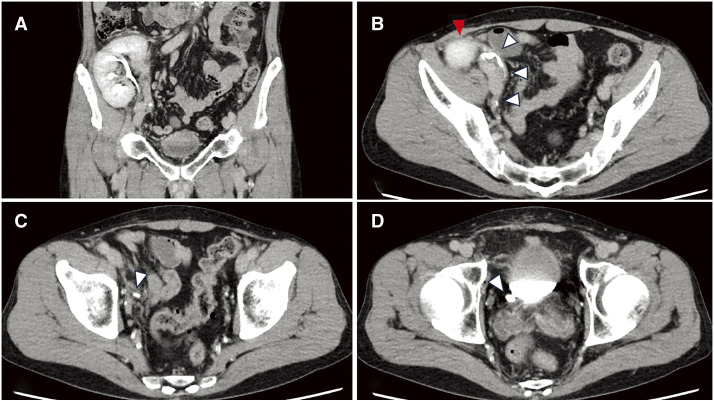
CE-CT findings. (**A**) The transplanted kidney is located in front of the iliac crest (coronal view). (**B**–**D**) The ureter (white arrowhead) runs medially, dorsally, and caudally from the transplanted kidney (red arrowhead) and enters the bladder (axial view). CE-CT, contrast enhanced CT

No inguinal hernia was detected on abdominal CT. Ureteral confirmation during the excretory phase revealed that the transplanted kidney was located in front of the right ilium. The transplanted ureter was placed into the bladder from the medial side of the transplanted kidney through the retroperitoneum (**Fig. 1A**–**1D**). The transplanted ureter ran sufficiently dorsal from the hernial orifice, and the extent of the peritoneal dissection area for TAPP could be secured.

#### Final diagnosis

The final diagnosis in the present case was ipsilateral right inguinal hernia after KT.

### Treatment

#### Strategy

Based on the CT findings, we decided that laparoscopic surgery with the TAPP procedure was feasible. We planned the surgical strategy as follows: a laparoscopic examination would be performed at the start of surgery to determine the position of the transplanted kidney and ureter, and we would switch to the anterior approach if the transplanted kidney and ureter were close to the hernial orifice.

#### Surgical findings

**Fig. 2:**
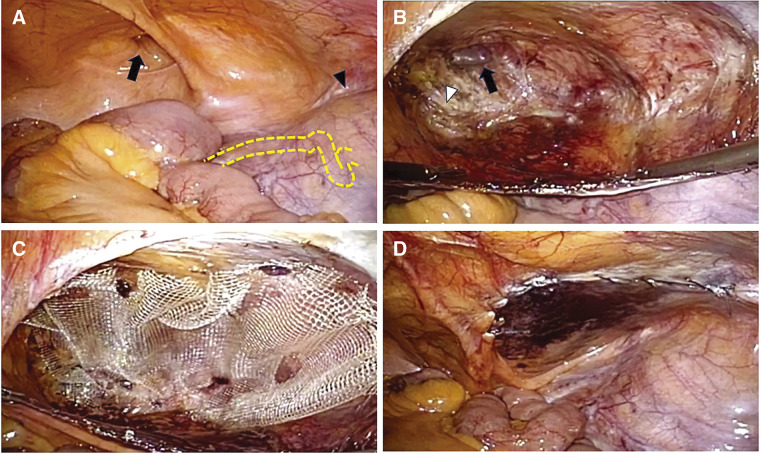
Intraoperative images. (**A**) Right inguinal hernia (black arrow), bulge of the transplanted kidney (arrowhead) on the right side, and peristalsis of the ureter (yellow dotted line) can be observed. (**B**) After dissection of the preperitoneal space, the hernial orifice (black arrow) and Cooper’s ligament (white arrowhead) can be observed. (**C**) Mesh is deployed in the anterior space of the peritoneum. (**D**) Peritoneal closure.

The patient was placed in the supine position under general anesthesia combined with a transversus abdominis plane block. A 5-mm trocar was inserted at the umbilical level using optical trocar insertion. After confirming intra-abdominal adhesions, two 5-mm trocars were placed at the level of the umbilicus in both the lateral abdomens. The inguinal region was observed, and a right inguinal hernia of M1 (EHS groin hernia classification) was found (**Fig. 2A**–**2D**).^7)^

The transplanted kidney and ureter were identified from the abdominal cavity, and the ureter was at least 4 cm away from the hernial orifice. Thus, peritoneal dissection and mesh deployment were possible. A peritoneal incision was placed 4 cm ventral to the hernial orifice using a high-incision peritoneal approach (HIPA),^8,9)^ and dissection was performed dorsally to completely invert the hernial sac. Cooper’s ligament, spermatic duct, and spermatic vessels were confirmed, and the myopectineal orifice (MPO) was preserved. On the dorsal side, the dissection was completed at a level that did not expose the transplanted ureter. After adequate dissection, a 10 × 15-cm TiLENE extralight mesh (Medical Leaders, Tokyo, Japan) was deployed into the preperitoneal space, and AbsorbaTack (Medtronic, Dublin, Ireland) was used to fix the mesh. The peritoneum was closed with continuous sutures.

#### Outcome and follow-up

The patient’s postoperative course was uneventful. No abnormal blood analysis findings were observed on the day after the surgery, and the patient was discharged on POD 2. No recurrence was observed at 21 months postoperatively.

### Case 2

#### Chief complaints

A 58-year-old woman with a right inguinal bulge was referred to our department.

#### Details of the present illness

The patient’s symptoms began immediately after KT, and gradually exacerbated to the point of pain.

#### The history of past illnesses

She had been introduced to dialysis at 52 years old due to kidney failure caused by a horseshoe kidney and underwent living-donor KT at 57 years old.

#### Physical examination findings

Her height was 143 cm, and her body weight was 38.8 kg. On physical examination, the abdomen was flat and soft, and a surgical scar (approximately 10 cm) from the Gibson incision used for KT was observed in the right lower quadrant. A bulge the size of an egg was noted in the right inguinal area while standing, spontaneously disappearing on lying down.

#### Laboratory examinations

A blood analysis showed BUN, creatinine, and eGFR values of 18 mg/dL, 1.18 mg/dL, and 37.3 mL/min/1.73 m^2^, respectively, suggesting mild kidney impairment. However, no inflammation or other abnormal findings were observed.

#### Imaging examinations

**Fig. 3:**
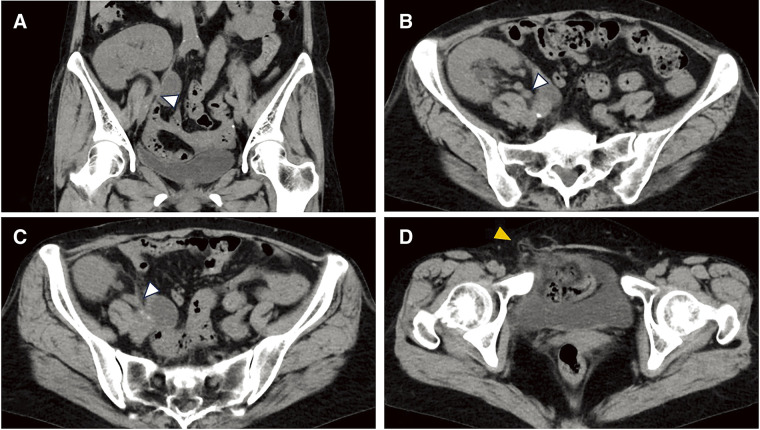
Plain CT findings. (**A**) Coronal view and (**B**–**D**) axial view: The ureter (white arrowhead) runs medially, dorsally, and caudally from the transplanted kidney, but its entry into the bladder is unclear. A direct hernia with fat content is observed (orange arrowhead).

Contrast-enhanced CT (CE-CT) was avoided due to the low eGFR, with plain CT performed instead. A direct hernia with fat content was observed in the right inguinal region. Because the transplanted ureter was not detected, the distance between the hernial orifice and the transplanted ureter was difficult to measure (**Fig. 3A**–**3D**).

#### Final diagnosis

The final diagnosis in the present case was an ipsilateral right inguinal hernia after KT.

### Treatment

#### Strategy

Based on the CT findings, we planned the surgical strategy as follows: a laparoscopic examination was performed to determine the location of the transplanted kidney and ureter, and the TAPP procedure would be performed if laparoscopic surgery seemed feasible. However, if the transplanted kidney or ureter was close to the hernial orifice, an anterior approach while checking the ureter with a laparoscope (hybrid method) would be performed.

#### Surgical findings

**Fig. 4:**
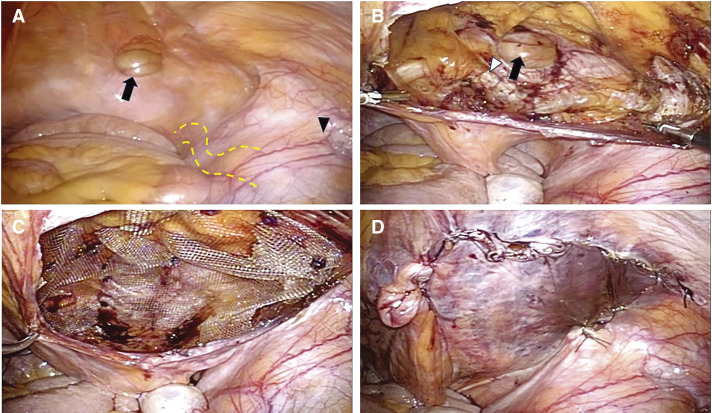
Intraoperative images. (**A**) Right inguinal hernia (black arrow), bulge of the transplanted kidney (arrowhead) on the right side, and peristalsis of the ureter (yellow dotted line) can be observed. (**B**) After dissection of the preperitoneal space, the hernial orifice (black arrow) and Cooper’s ligament (white arrowhead) can be observed. (**C**) Mesh is deployed in the anterior space of the peritoneum. (**D**) Peritoneal closure.

Trocar placement was the same as in Case 1, and laparoscopic findings revealed a right inguinal hernia of M1 (**Fig. 4A**–**4D**). Because a sufficient distance to the peritoneal dissection and mesh deployment between the transplanted ureter and the hernial orifice was observed, we determined that TAPP with HIPA could be performed. The MPO exposure and mesh placement were performed in the same manner as in Case 1.

#### Outcome and follow-up

The postoperative course was uneventful. No abnormal blood test results were observed. The patient was discharged on POD 2. No recurrence was observed at 17 months postoperatively.

## DISCUSSION

Inguinal hernias after KT seem to be very rare, as there is no statistical report about their incidence.^3)^ There are several reasons for this. First, the actual complication rate is low. Second, the present patients were only followed up because of concerns about injury to the transplanted ureter during herniorrhaphy or the immunocompromised state caused by immunosuppressive drugs. However, surgery is considered the first choice because there have been reports of transplanted ureteral obstruction due to prolapse from the hernial orifice.^4,10,11)^

There are 2 herniorrhaphy procedures: laparoscopic and anterior. Most KT recipients have undergone herniorrhaphy using the anterior approach (mainly the Lichtenstein method) because of the risk of transplanted ureteral injury with laparoscopic surgery.^2,3)^ It is believed that the anterior approach can be performed even if the transplanted ureter runs around the dissection area during laparoscopic herniorrhaphy. In addition, many articles recommend the Lichtenstein procedure because it does not require manipulation of the preperitoneal space. However, there are some concerns about transplanted ureteral injury if the position of the transplanted ureter is not confirmed (e.g., direct injury of the transplanted ureter, occlusion by traction with a mesh fixation suture, and compression by mesh when the transplanted ureter protrudes through the hernial orifice). In fact, a few reports have described obstructive uremia after the Lichtenstein procedure^5,6)^; thus, ureteral injury or obstruction should be considered even when this procedure is performed.

The TAPP procedure has a great advantage over the anterior approach in that the transplanted kidney and ureter can be visualized laparoscopically, avoiding blind dissection, which is a problem with the anterior approach. However, it is necessary to be careful about transplanted ureteral injury and stricture caused by compression from the mesh because there are severe adhesions or fibrotic changes that exist around the dissection area. The choice of mesh material is important to minimize the negative effects of the mesh. In recent years, large-pore meshes have been reported to have less shrinkage and less tissue damage; therefore, it is necessary to select such meshes.^12)^

To ensure safe laparoscopic herniorrhaphy, preoperative studies evaluating the transplanted ureteral course and the safe area for peritoneal dissection are important. Although preoperative CT for inguinal hernia is not generally needed, CE-CT for KT recipients in the excretory phase (CT urography) may be useful to confirm the position of the transplanted ureter. However, CE-CT is difficult in some cases because of kidney dysfunction, so plain CT and exploratory laparoscopy should be performed in these cases.

The main advantage of laparoscopic surgery for inguinal hernias, including TAPP and laparoscopic totally extraperitoneal surgery, is the complete coverage of MPO.^13,14)^ TAPP can evaluate the location of the transplanted ureter and kidney from the abdominal cavity during MPO dissection. Therefore, this procedure is useful in kidney transplant patients. The mesh positioning is defined by the Golden Rule to cover the MPO with a minimum overlap of 3 cm or more.^14)^ In general condition cases, the overlap margin should be determined based on the MPO. On the other hand, in the kidney transplant recipients, the lateral–posterior area requiring dissection to deploy the mesh is traversed by the transplanted kidney and ureter. This increases the injury risk of transplanted kidney and ureter. Therefore, we intentionally limited the lateral–posterior dissection, with the margin measured at least 4 cm from the direct inguinal hernia defect, because this distance is considered sufficient to achieve overlap with a safety margin. In our cases, both types of hernia were direct inguinal hernias. Thus, it was possible to secure sufficient space for dissection (particularly in the lateral–posterior region) required for mesh deployment. On the other hand, in cases of large indirect inguinal hernia, especially L3 hernia, securing the dissection area (less than 4 cm) may be difficult due to the transplanted kidney, ureter, or severe adhesions, and the risk of other organ injury increases. Consequently, alternative approaches, such as hybrid or anterior approaches, should be considered.

We used the HIPA approach in our cases, which involves incising the peritoneum approximately 4 cm above the hernial orifice.^8)^ This procedure has been reported for inguinal hernia after radical prostatectomy, with the advantage of avoiding severe adhesions from radical prostatectomy during peritoneal dissection.^9)^ For the same reason, the HIPA approach can be useful for ipsilateral inguinal hernia after KT.

Tension-free repair using mesh is the mainstay of herniorrhaphy and is an appropriate procedure for inguinal hernia patients after KT. In Japan, the mesh plug method is used in most anterior approaches, and it is expected that this method will be used more frequently in kidney transplant patients. However, Ortiz et al. reported a case of transplanted ureteral dysfunction caused by inserting a plug placed near the transplanted ureter due to strong adhesions and blood flow disturbance.^15)^ Thus, it is necessary to ensure a sufficient distance between the mesh and the ureter or consider other procedures for herniorrhaphy.

In contrast, mesh placement in laparoscopic herniorrhaphy tends to be closer than that in the anterior approach. Thus, dissection of the anterior peritoneal space should be limited so that the ureter is not exposed. When the distance from the hernial orifice to the transplanted ureter is close, typically less than 3 cm, a hybrid approach (preferably laparoscopic plus Lichtenstein herniorrhaphy) should be considered to avoid injury to the transplanted organs. The advantages of the hybrid approach are as follows: first, laparoscopic visualization of the ureteral course avoids excessive traction or accidental ligation of the ureter; second, complete hernia fixation using mesh can be confirmed.

Thus, TAPP is a suitable treatment for inguinal hernia after KT in terms of confirmation of the transplanted ureteral course, secure exposure of the MPO, and mesh deployment. However, it is important to confirm whether or not mesh deployment is feasible on CE-CT, including the position of the transplanted ureter, location of the transplanted kidney, and distance from the hernial orifice to the transplanted ureter.

When CE-CT cannot be performed in KT recipients, plain CT should be performed to evaluate the location of the transplanted kidney and ureter as accurately as possible. Exploratory laparoscopy should be performed to evaluate the adequate area for mesh deployment at the time of surgery.

Kidney transplant recipients require long-term follow-up after herniorrhaphy due to their compromised immunological and physiological status compared to the general population. For example, the risk of recurrence is high because adhesion between the implanted mesh and the surrounding tissue is poor. The administration of immunosuppressants also carries a long-term risk of mesh infection. Additionally, caution is required regarding delayed ureteral complications, such as ureteral compression or stricture caused by mesh migration or delayed adhesions.

## CONCLUSIONS

A preoperative evaluation of the transplanted kidney and ureter by CT (CE-CT, if possible) is the most important aspect of the surgical strategy for KT recipients with groin hernia. The TAPP procedure is considered the most appropriate surgical procedure when an adequate distance between the hernial orifice and the transplanted ureter can be confirmed.

## DECLARATIONS

### Availability of data and materials

The data that support the findings of this study are available from the corresponding author upon reasonable request.
